# Molecular glucose imaging reveals functional brain reconfiguration by subthalamic deep brain stimulation in Parkinsonian rats

**DOI:** 10.1186/s40035-025-00523-3

**Published:** 2025-12-02

**Authors:** Jiazhi Chen, Ningfei Li, Muthuraman Muthuraman, Nengxing Liang, Jens Volkmann, Takahiro Higuchi, Chi Wang Ip

**Affiliations:** 1 Department of Neurology, University Hospital of Würzburg, Josef-Schneider-Straße 11, 97080 Würzburg, Germany; 2 Movement Disorders and Neuromodulation Unit, Department of Neurology, Charité-Universitätsmedizin Berlin, Corporate Member of Freie Universität Berlin and Humboldt-Universität zu Berlin, Charitéplatz 1, 10117 Berlin, Germany; 3 Department of Nuclear Medicine and Comprehensive Heart Failure Center, University Hospital of Würzburg, Oberdürrbacher Straße 6, 97080 Würzburg, Germany

**Keywords:** Reverse-translation, Deep brain stimulation, Alpha-synuclein, Positron emission tomography, Glucose metabolism, Electrode modeling, Electric field, Volume of tissue activated

## Abstract

**Background:**

In order to elucidate the neuromodulatory mechanisms underlying therapeutic subthalamic deep brain stimulation (DBS), we here reverse-translate a methodological pipeline that integrates neurostimulation effect parameterization and molecular imaging.

**Methods:**

^18^F-fluorodeoxyglucose positron emission tomography was performed in a human-mimicking A53T alpha-synuclein Parkinson’s disease rat model and in control rats under both stimulation ON and OFF conditions, with additional CT scans acquired for each rat. Patient-derived approaches—including electrode modeling, electric field estimation, and volume of tissue activated measurement—were applied to assess stimulation effects at the stimulation spot.

**Results:**

We revealed consistent hypometabolism in the ipsilateral subthalamic nucleus, substantia nigra, zona incerta, cerebellum, and entopeduncular nucleus, alongside hypermetabolism in the ipsilateral lateral caudate putamen and globus pallidus externus in A53T rats at the OFF condition. Subthalamic DBS improved motor dysfunction and induced specific metabolic responses that differentiated from controls, including increased metabolism in the ipsilateral subthalamic nucleus, substantia nigra, and zona incerta, and decreased metabolism in the bilateral primary motor and somatosensory area, lateral caudate putamen, and contralateral secondary motor area.

**Conclusions:**

Therapeutic subthalamic DBS activates the target region and modulates global brain function by restoring OFF-state hypometabolism in the ipsilateral subthalamic–substantia nigra loop and by reducing metabolic activity in the bilateral cortico-striatal circuitry. A reverse-translational pipeline is established to study stimulation-induced modulation of brain function, integrating a novel positron emission tomography template aligned with the Waxholm space of Sprague–Dawley rats.

**Supplementary Information:**

The online version contains supplementary material available at 10.1186/s40035-025-00523-3.

## Background

High-frequency subthalamic deep brain stimulation (STN-DBS) is an established therapy for alleviating the cardinal motor symptoms of Parkinson’s disease (PD) [1]. However, its neural mechanisms are not fully understood. It has been suggested that the therapeutic effects of STN-DBS are mediated through modulation of the cerebral brain activity, rather than merely the local effects on the stimulated target [2, 3]. Studies using ^18^F-fluorodeoxyglucose positron emission tomography (FDG-PET)—which measures cerebral glucose metabolism and reflects integrated synaptic [4] and neural activities [5]—have been extensively used to explore the neuronal mechanisms of STN-DBS in PD [6]. However, existing studies primarily focused on the effects of bilateral STN-DBS [6], which may obscure our understanding of how unilateral STN-DBS influences bilateral hemispheres.

Differences in disease severity lead to varying symptoms and distinct neurodegeneration-related brain activity [7], which may result in diverse functional responses to STN-DBS. There is significant interest in first understanding the intrinsic functional properties in the OFF-stimulation state and subsequently examining their modulation by DBS. However, most studies investigating DBS-induced activity changes in PD patients have primarily relied on comparisons between the OFF- and ON-stimulation states [6]. While this approach captures the net effects of DBS, it lacks a detailed characterization of the OFF-state brain activity. A key limitation arises from the challenge of precisely identifying disease-related activity in the OFF-stimulation PD state when compared to healthy controls without electrode implantation or pre-implantation PD subjects [8]. This is because electrode implantation itself may induce local or global metabolic changes [8, 9]. As a result, this limitation hinders a comprehensive understanding of the circuit mechanisms underlying DBS.

Reverse translation, also known as ‘bedside-to-benchtop’ research, initially highlights a backward approach to uncover underlying mechanisms based on clinical experiences and observations [10]. Recently, methodological reverse translation has also been emphasized, calling for closer alignment between preclinical approaches and patient studies [11]. In our previous study, we adapted the Lead-DBS toolbox—a widely used platform for analyzing human DBS data—to establish an electrode modeling pipeline in healthy rats [12]. This approach enables three-dimensional electrode localization and facilitates quantitative assessment of stimulation effects on target regions. While the Lead-DBS toolbox is well validated in clinical studies and healthy rats, its application in PD rat models has not yet been established.

In this study, we leverage the advantages of the unilaterally lesioned PD animal model by using the AAV1/2-A53T-alpha-synuclein (α-syn) rat model—which closely mimics human PD, characterized by nigrostriatal deficiency, Lewy body pathology, and motor impairments [13, 14]—and conducted unilateral STN-DBS in the A53T rats and controls (empty vector, EV). We combined FDG-PET signal analysis with a parameterized DBS profile—including three-dimensional electrode modeling, electric field (E-field) estimation, and volume of tissue activated (VTA) calculations—to investigate STN-DBS-induced local and brain-wide modulation. This reverse-translational approach integrates clinical tools into preclinical research, enhancing our ability to investigate DBS mechanisms and improve the translational relevance of experimental findings [11]. The parameterized DBS profiles open new avenues for data integration and comparison across different animal cohorts and research centers.

## Methods

### Experimental timeline

**Fig. 1:**
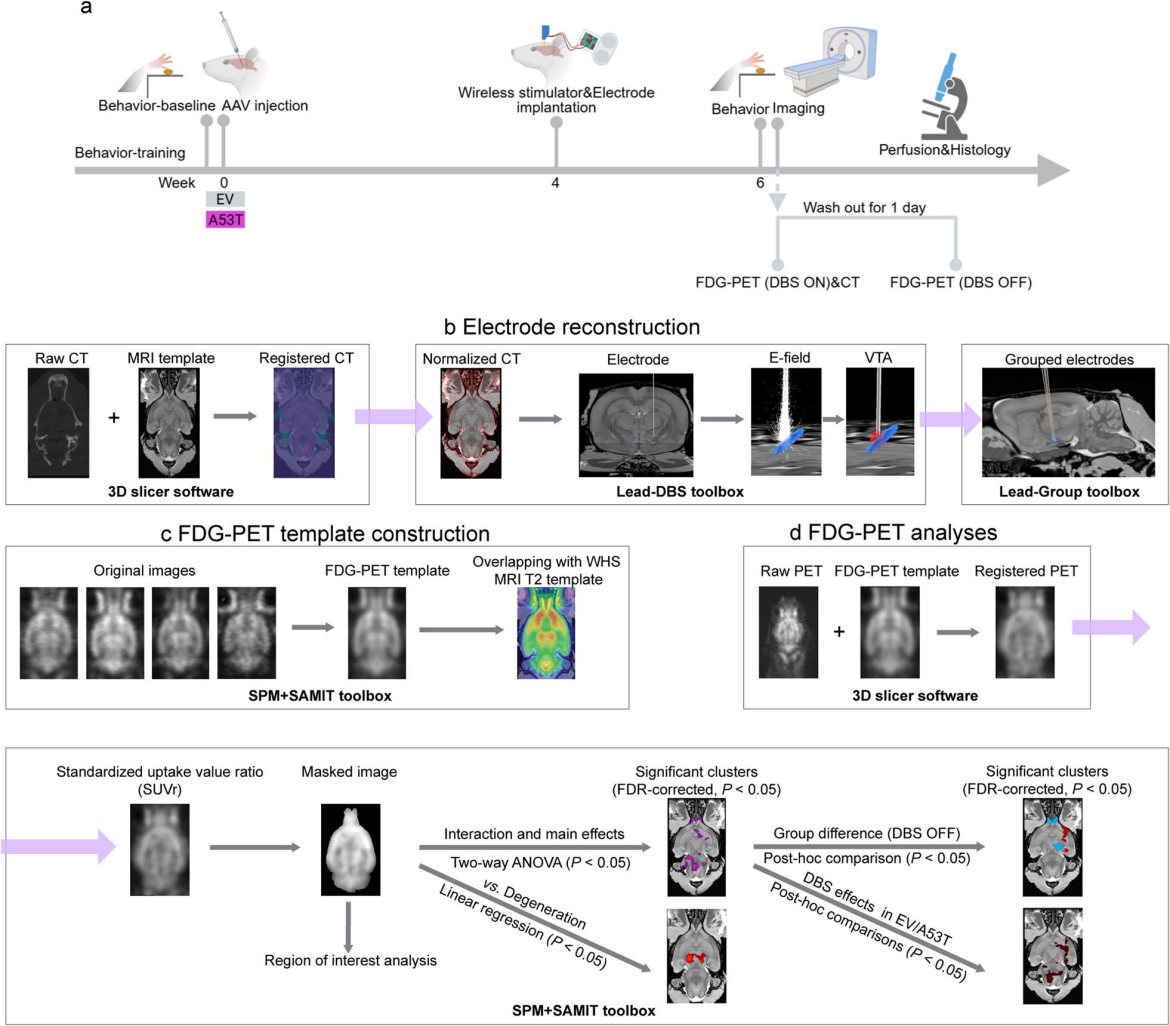
Experimental timeline and imaging data processing. **a** Schematic of the experimental timeline highlighting key procedural milestones (created with BioRender). **b**–**d** Diagrams illustrating the main procedures: **b** electrode reconstruction, **c** FDG-PET template construction, and **d** PET image analysis

The experimental timeline is illustrated in Fig. 1a. Rats underwent a 16-day, single pellet reaching task training period, followed by baseline recordings. Rats were assigned to either the EV or A53T group for surgery. Four weeks after viral injection, monopolar DBS electrodes and wireless stimulators were implanted. Two weeks after implantation, stimulators were programmed, and single pellet reaching task recordings were performed under the ON and the OFF states, respectively. Subsequent imaging procedures included FDG-PET scan during the ON-stimulation state after 12 h of stimulation, and a CT scan in the OFF state. After a 1-day washout period, another FDG-PET scan was performed in the DBS-OFF state. One day after the final imaging session, rats were perfused, and histological procedures were performed.

### Rats

Sixteen adult male Sprague–Dawley rats were obtained from Charles River Laboratories (Sulzfeld, Germany). Four rats were used to construct the FDG-PET template, while the remaining 12 rats underwent the main experimental timeline, including behavioral training, surgeries, FDG-PET/CT scans with or without DBS, and final histological procedures. All rats were housed at the animal facility of the Center for Experimental Molecular Medicine, University of Würzburg, Germany, and maintained under standard conditions, including a temperature of 21 °C and a 12-h dark–light cycle. Animal experiments adhered to all applicable international, national, and institutional guidelines for the care and use of animals. Ethical approval was obtained from the local authorities at the Regierung von Unterfranken, Germany.

### Single pellet reaching task

Single pellet reaching task procedures are described in Additional file 1: Supplementary materials and methods.

### AAV injection

At week 0, 12 rats received stereotactical injection into the substantia nigra pars compacta (SNc) contralateral to the dominant forepaw (5 rats left-injected, 7 rats right-injected), as determined by the single pellet reaching task training. Briefly, 2.0 µl of empty AAV1/2 virus (EV group, *n* = 5 rats) or 2.0 µl of AAV1/2 expressing human mutated α-syn (A53T group, *n* = 7 rats), both at a concentration of 2.5 × 10^12^ genomic particles (gp)/mL, were injected using the following coordinates: − 5.2 mm anteroposterior and ± 2.0 mm mediolateral from bregma; − 7.4 mm dorsoventral from dura [15]. The injection was conducted at a speed of 0.2 μL/min; the syringe was left in place for 5 min to allow diffusion of the solution within the surrounding brain tissue and was then retreated from the target at a speed of 1.0 mm/min.

### Implantation and programming

Four weeks after AAV injection, a monopolar quartz-glass-insulated platinum–tungsten fiber microelectrode (Thomas RECORDING GmbH, Giessen, Germany) was stereotaxically implanted into the STN (− 3.6 mm anteroposterior and ± 2.5 mm mediolateral from bregma, − 7.7 mm dorsoventral from dura [15]), ipsilateral to the viral injection side. A ground electrode was implanted over the cerebellum. After implanting the electrodes, a wireless stimulator (as described by [16], Thomas RECORDING GmbH) was placed on the posterior aspect of the cerebellum and secured in position using dental cement. Notably, the stimulator was carefully placed to ensure no overlap with brain tissue, and was solely connected to the headset using cement, avoiding any direct contact with muscle or skin. Afterward, the wires extending from the DBS electrode were connected to the stimulator. Finally, all wires and the electrode pin were stabilized by embedding them with UV dental cement (Filtek Supreme XTE Composite, 3M Deutschland GmbH) and dental acrylic cement (Paladur, Kulzer GmbH, Germany). Only the programming socket remained exposed but was also secured in place. Two weeks after the implantation, the stimulation parameters were configured with a rectangular pulse shape (charge-balancing phase), with a pulse width of 60 μs and a frequency of 130 Hz. The amplitude was set at 20% below the threshold for inducing side effects such as orofacial, axial, or forepaw dyskinesia [14]. As the programming interface (Silicon Laboratories IDE, version 5.50; Silicon Labs, Austin, TX) permitted current selection in 5 µA increments, the actual stimulation current applied was set to the largest 5 µA multiple not exceeding the calculated value. Basic electrode properties and DBS parameters are summarized in Table S1.

### FDG-PET/CT scan and image analyses

FDG-PET scans were conducted using a small-animal PET system (Inveon; Siemens Medical Solutions, Knoxville, TN). Rats were subjected to overnight fasting with free access to water. Fifty-three minutes after intraperitoneal administration of ^18^F-FDG (~ 45 MBq), rats were anesthetized with 0.3% isoflurane in oxygen for 7 min and maintained under anesthesia with 2% isoflurane in oxygen throughout the 10-min static PET and subsequent CT scan. PET images were reconstructed using Fourier transformation to generate dynamic images, employing a 3D ordered-subset expectation maximization algorithm. CT scan was conducted using a U-SPECT system (U-SPECT5/CT E-Class; MILabs, Utrecht, The Netherlands). The pipelines for CT analysis, FDG-PET template construction, and PET analysis are illustrated in Fig. 1b–d.

#### Electrode localization and E-field estimation

The DBS electrodes were localized as previously described [12]. CT images from the left-injected rats were flipped (left to right) so that the right side represents the injected and implanted hemisphere for all rats, ensuring consistent comparisons across animals and accurate group-level analyses. CT images were first linearly co-registered (12 degrees of freedom ‘*affine*’ transformation) to the downsampled MRI T2 template (0.05 × 0.05 × 0.05 mm) in the Waxholm Space (WHS) for the Sprague–Dawley rats (*WHS_SD_rat_T2star_v1.01*, https://www.nitrc.org/projects/whs-sd-atlas) using 3D Slicer [17]. The success of the co-registration was visually examined and approved with the Lead-DBS software (Version 2.6) [18]. The electrode trajectories were then reconstructed using the ‘*manual*’ method in Lead-DBS by marking the tip (contact) and a point on the trajectory in the co-registered CT images with high contrast. Finally, the rendered electrode was visualized in WHS [19].

A current (µA)-dependent E-field estimation was performed in Lead-DBS using the built-in SimBio/FieldTrip pipeline with default settings [20]. Generated E-fields were further thresholded at 0.2 V/mm, based on which binarized VTAs were also created [21]. The weighted score of E-field–STN overlap was calculated by multiplying the binary STN image with the non-binary E-field and summing the resulting voxel values [18]. The distance between the nearest voxel of the VTA and the STN centroid was calculated by identifying the closest voxel in the VTA to the centroid of the STN and measuring the Euclidean distance between their coordinates in three-dimensional space.

#### FDG-PET template construction

The detailed procedures have been described previously [22]. FDG-PET images from control rats (*n* = 4) were utilized and the main procedures were conducted in the statistical parametric mapping (SPM, SPM12, Wellcome Department of Cognitive Neurology, University College London, UK) and SAMIT toolbox [22]. A down-sampled (0.2 × 0.2 × 0.2 mm) MRI T2 template and corresponding mask for Sprague–Dawley rats were utilized for reference. After construction, the FDG-PET template was integrated into WHS in SAMIT toolbox and served as a reference for spatial normalization of FDG-PET images.

#### FDG-PET image preprocessing

FDG-PET images from left-injected rats were flipped (left to right), consistent with the CT analysis. FDG-PET images were linearly co-registered (7 degrees of freedom ‘*rigid* + *scale*’ + 12 degrees of freedom ‘*affine*’ transformation) to the template constructed above, using 3D Slicer [17]. The successful completion of co-registration was visually verified and approved in 3D slicer. Using the SAMIT toolbox [22], the standardized uptake value ratio (SUVr) for FDG was calculated by dividing the values of a single voxel by the mean value of the whole brain. A binary mask of the whole brain was then used to exclude the extracerebral areas.

#### SPM analyses

Voxel-wise statistics were conducted using SPM12 (Wellcome Department of Cognitive Neurology, University College London, UK). A voxel-wise flexible factorial design with a two-way ANOVA was employed to identify the interaction and main effects. To assess the metabolic differences between OFF-stimulation EV and A53T rats, and the effect of STN-DBS in both EV and A53T rats, post-hoc tests were subsequently performed by directly setting the t-contrasts in SPM using the same input dataset. Linear regression analyses were utilized to explore the relationship between nigrostriatal denervation and cerebral metabolism. For all SPM analyses, cluster-level significance was determined by thresholding the *P*-value at 0.05, followed by false discovery rate (FDR) correction at *P* < 0.05. Motor regions covered by significant clusters were identified manually through overlay with the WHS atlas (*WHS_SD_rat_atlas_v4.01*, https://www.nitrc.org/projects/whs-sd-atlas) and the MRI T2 template in MRIcroGL.

#### Region of interest analysis

The STN mask was constructed using a down-sampled (0.2 × 0.2 × 0.2 mm) WHS atlas of the Sprague–Dawley rat, and the mean SUVr values of the STN were then calculated in MATLAB (Version 2022b, MathWorks, Natick, MA) by performing voxel-wise multiplication of the mask with PET images, followed by calculation of the mean values within the masked area.

### Histology and immunohistochemistry

Procedures for histology and immunohistochemistry are described in Additional file 1: Supplementary materials and methods.

### Statistical analysis

All statistics (except voxel-wise analyses) were conducted using GraphPad Prism (Version 9.3.1, GraphPad Software, San Diego, CA). Normality was assessed using the Shapiro–Wilk test. Unpaired *t*-tests (normal distribution) or Mann–Whitney test (non-normal distribution) was employed to compare differences between EV and A53T groups across parameters, including tyrosine hydroxylase-positive (TH^+^)/Nissl^+^ SNc neuron number, striatal dopaminergic fiber density, weighted score of E-field–STN overlap, Euclidean distance between the nearest voxel of VTA and STN centroid, behavioral score, and behavioral kinematics. Pearson (normal distribution) or Spearman (non-normal distribution) correlation analysis was utilized to explore the association between ‘Euclidean distance between the nearest voxel of VTA to STN centroid/E-field–STN overlap’ and ‘metabolic changes in stimulated-STN/Hexokinase (HK)1 intensity’. Paired *t*-tests (normal distribution) or Wilcoxon matched-pairs signed rank test (non-normal distribution) was used to compare FDG uptake in the STN, behavioral score, and behavioral kinematics pre- versus post-DBS in A53T rats. *P* < 0.05 was considered as statistically significant.

## Results

### Electrode implantation and PD model assessments

**Fig. 2:**
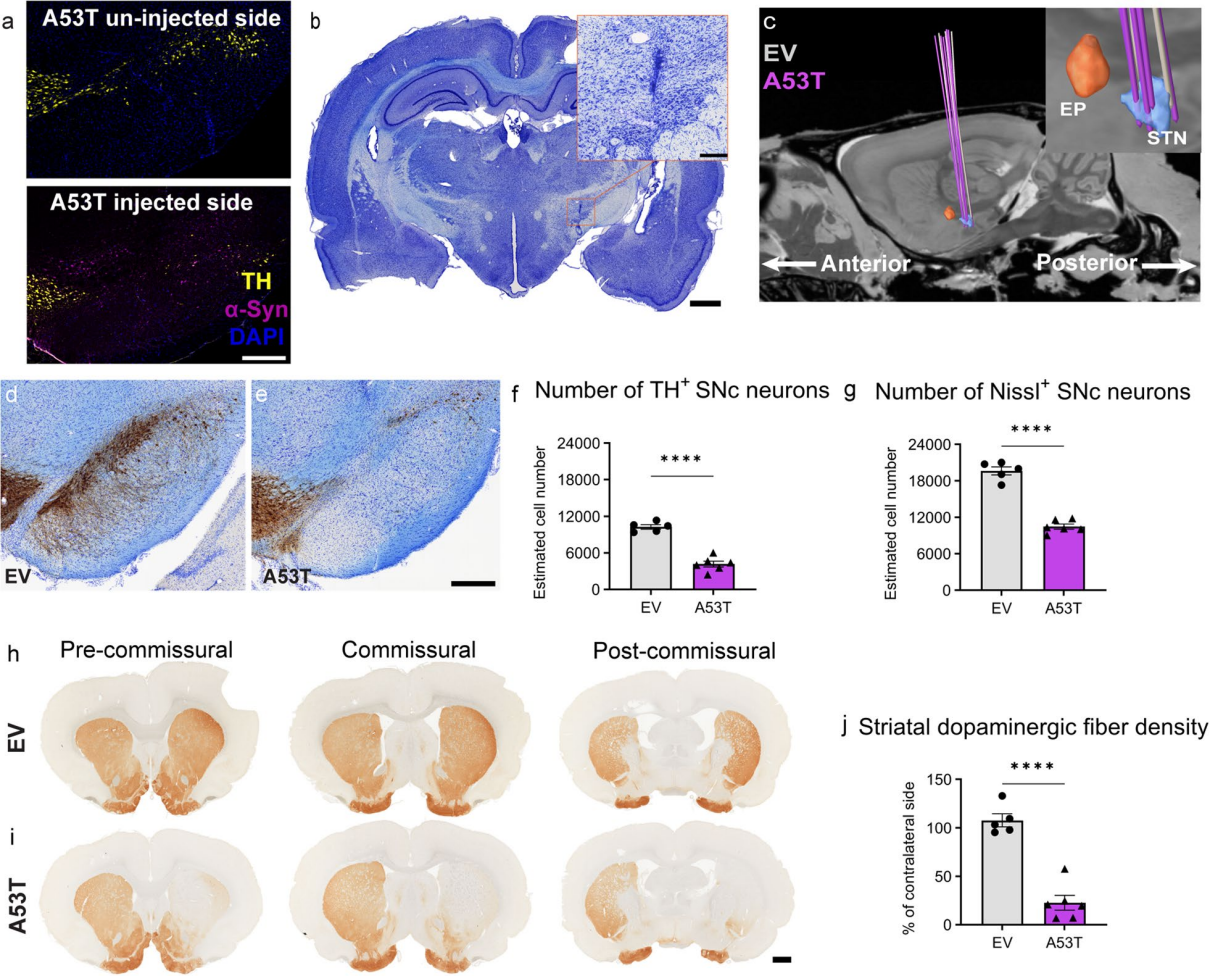
Electrode localization and histology. **a** Immunofluorescence staining of TH^+^ neurons (yellow), α-syn (magenta), and DAPI (blue) for SNc sections of A53T rats (scale bar: 400 μm). **b** Nissl staining of the STN section (scale bar: 1000 μm), with a magnified view of the electrode track shown in the upper right corner (scale bar: 200 μm). **c** Group visualization of the electrode reconstruction in sagittal view of all EV (grey, *n* = 5) and A53T (pink, *n* = 7) rats. **d**, **e** TH-Nissl^+^ immunohistochemical staining for SNc sections from EV (**d**) and A53T (**e**) rats. Scale bar: 400 µm. **f**, **g** TH^+^ and Nissl^+^ neuron numbers in the SNc of EV (*n* = 5) and A53T (*n* = 6) rats. **h**, **i** TH^+^ immunohistochemical staining for striatal sections (left: pre commissural; middle: commissural; right: post-commissural) of EV (**h**) and A53T (**i**) rats; scale bar: 1000 µm. **j** Striatal dopaminergic fiber density of EV (*n* = 5) and A53T (*n* = 6) rats. Bar graphs are presented as individual symbols with mean ± SEM. Unpaired *t*-tests (**f**, **g**, **j**); ****P < 0.0001

To determine the success of electrode implantation into the STN, we performed both conventional Nissl staining and electrode modeling using the Lead-DBS toolbox. The expression of α-syn in dopaminergic neurons was evaluated by immunofluorescence staining on SNc sections in A53T rats (Fig. 2a). A53T rats with consistent α-syn distribution within the SNc area were included for group comparisons with EV. One A53T-injected rat with poor A53T spreading was excluded for comparisons with EV. All rats met the Nissl staining and image-based accuracy requirements (Fig. 2b, c). In contrast to EV controls, A53T rats demonstrated TH^+^ neuron loss in the SNc (Fig. 2d, e). Compared to EV rats (*n* = 5), the A53T rats (*n* = 6) exhibited a ~ 60% reduction of TH^+^ dopaminergic neurons (4175 ± 487.7 vs*.* 10,272 ± 357.5; unpaired *t*-tests, *t*_(9)_ = 9.703, *P* < 0.0001; Fig. 2f) and a ~ 47% decrease in the Nissl^+^ total neurons (10,494 ± 426.6 vs. 19,644 ± 671.0; unpaired *t*-tests, *t*_(9)_ = 11.92, *P* < 0.0001; Fig. 2g) in the SNc. TH^+^ staining of the striatum exhibited a decline in A53T rats (*n* = 6) compared to the EV controls (*n* = 5) (Fig. 2h, i), with a significant ~ 79% decrease in dopaminergic fiber density in the striatum (22.83% ± 7.621% vs. 107.7% ± 6.787%; unpaired *t*-tests, *t*_(9)_ = 8.143, *P* < 0.0001; Fig. 2j).

### Local modulation by STN-DBS in A53T rats

**Fig. 3:**
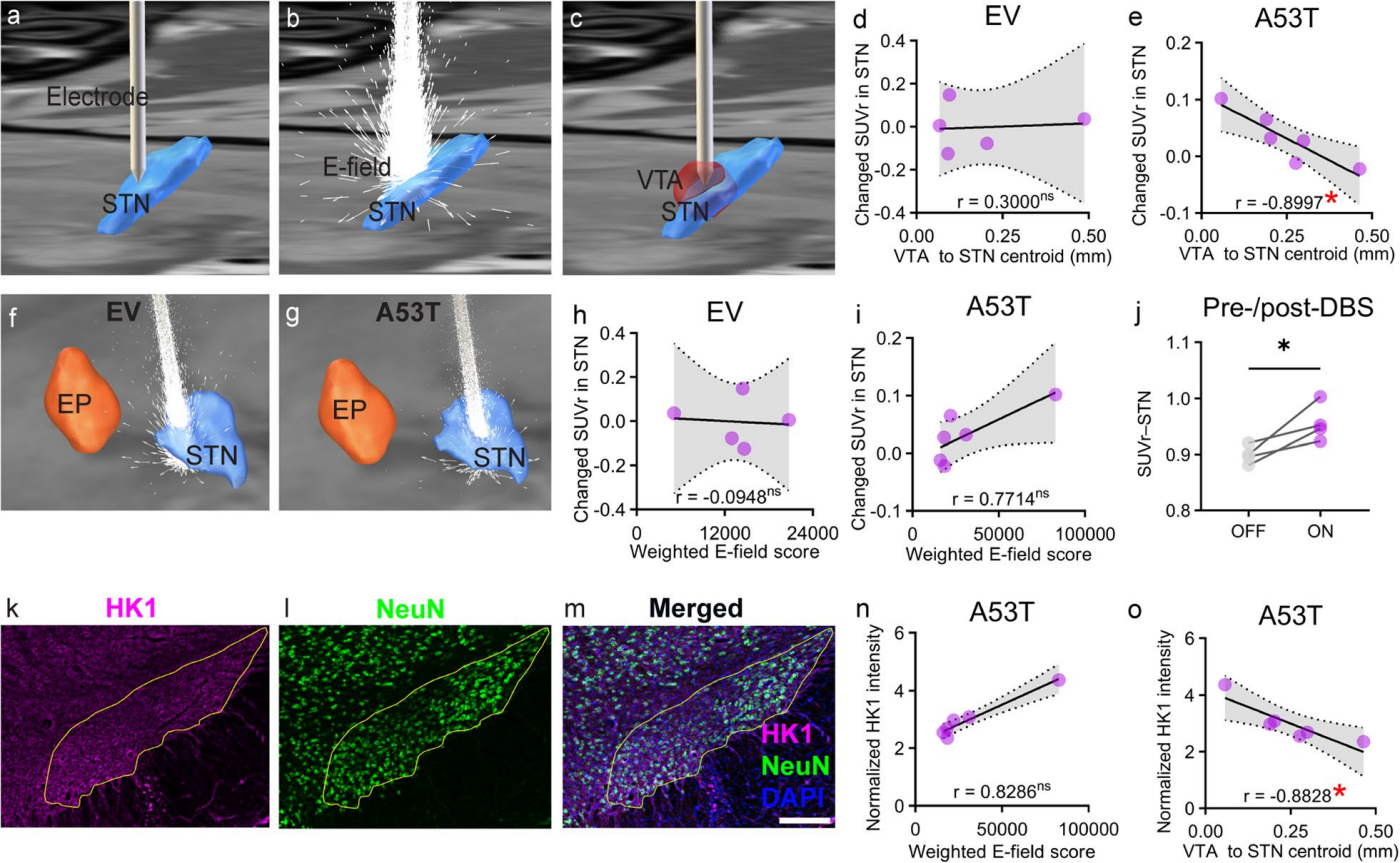
Metabolic impact of STN-DBS in A53T rats. **a**–**c** Representative images of DBS electrode localization (**a**), E-field distribution (**b**), and VTA (**c**). All relevant elements are annotated and overlaid on the Waxholm MRI T2 template in the transverse view. **d**, **e** Correlation analysis of Euclidean distance (nearest voxel of VTA to STN centroid) with SUVr changes (SUVr_DBS-ON_–SUVr_DBS-OFF_) in the STN of EV (**d**) and A53T rats (**e**). **f**, **g** Representative images of E-field distribution in EV (**f**) and A53T (**g**) rats. **h**, **i** Correlation analysis of the weighted score of E-field–STN overlap with SUVr changes post-DBS in the STN of EV (**h**) and A53T (**i**) rats. **j** Mean FDG uptake in the STN pre- and post-DBS in A53T rats (paired *t*-test, *P < 0.05). **k**–**m** Immunofluorescence staining of HK1 (magenta), NeuN (green), and DAPI (blue) for STN sections of A53T rats (scale bar: 200 μm). The border of STN area is outlined in yellow for reference. **n**, **o** Correlation analysis of the weighted score of E-field–STN overlap and the Euclidean distance (nearest voxel of the VTA to the STN centroid) with HK1 signal intensity in the STN of A53T rats. Pearson or Spearman correlation coefficients (*r* values) and statistical significance levels (*P < 0.05; ^ns^*P* > 0.05) are reported, with individual symbols representing data points

To elucidate the effects of STN-DBS on the target area in both EV and A53T rats, we performed three-dimensional electrode reconstruction (Fig. 3a), E-field (Fig. 3b), and VTA (Fig. 3c) calculations using the Lead-DBS toolbox. Notably, we discovered a strong negative correlation between the distance (nearest voxel of VTA to the STN centroid) and the changes in STN FDG uptake post-DBS in A53T rats (*n* = 6; Pearson *r* = − 0.8997, *P* < 0.05) rather than the EV rats (*n* = 5; Spearman *r* = 0.3, *P* > 0.05) (Fig. 3d, e). We further calculated the E-field in both EV and A53T rats (Fig. 3f, g), followed by calculation of a weighted score based on the E-field–STN overlap. No significant correlation was observed between the weighted score of the STN E-field and the FDG uptake within STN after DBS in both EV (*n* = 5; Pearson *r* = − 0.0948, *P* > 0.05) and A53T rats (*n* = 6; Spearman *r* = 0.7714, *P* > 0.05) (Fig. 3h, i). These results showed that an electrode placement within the STN centroid resulted in the strongest FDG uptake upon stimulation in A53T rats. We next included the rats (*n* = 4) that showed positive SUVr changes after DBS and performed a paired *t*-test at the region-of-interest level to examine whether the mean FDG uptake in the ON-stimulation STN differed significantly from those in the OFF-stimulation condition. The results indicated that the mean SUVr was significantly higher in the DBS ON state compared to the DBS OFF state (*n* = 4; 0.8994 ± 0.008 vs. 0.9562 ± 0.017; *t*_(3)_ = 3.217, *P* < 0.05; Fig. 3j), confirming the STN hypermetabolism after stimulation in the included A53T rats.

HK1 immunofluorescence staining further validated the effects of DBS on STN metabolism in A53T rats (Fig. 3k–m). While the normalized HK1 intensity in the STN did not show a significant correlation with the weighted E-field–STN overlap score (*n* = 6; Spearman *r* = − 0.8286, *P* > 0.05), it was significantly negatively correlated with the Euclidean distance between the nearest voxel of the VTA and the STN centroid (*n* = 6; Pearson *r* = − 0.8828, *P* < 0.05) (Fig. 3n, o). Together with the FDG-PET findings, these results validated local metabolic activation induced by STN-DBS in A53T rats. Rats that exhibited STN activation following DBS were expected to show reliable stimulation effects across brain-wide regions; therefore, only these animals (*n* = 4) were included in the ON-stimulation A53T group for subsequent analyses.

### Brain-wide alterations in A53T rats

**Fig. 4:**
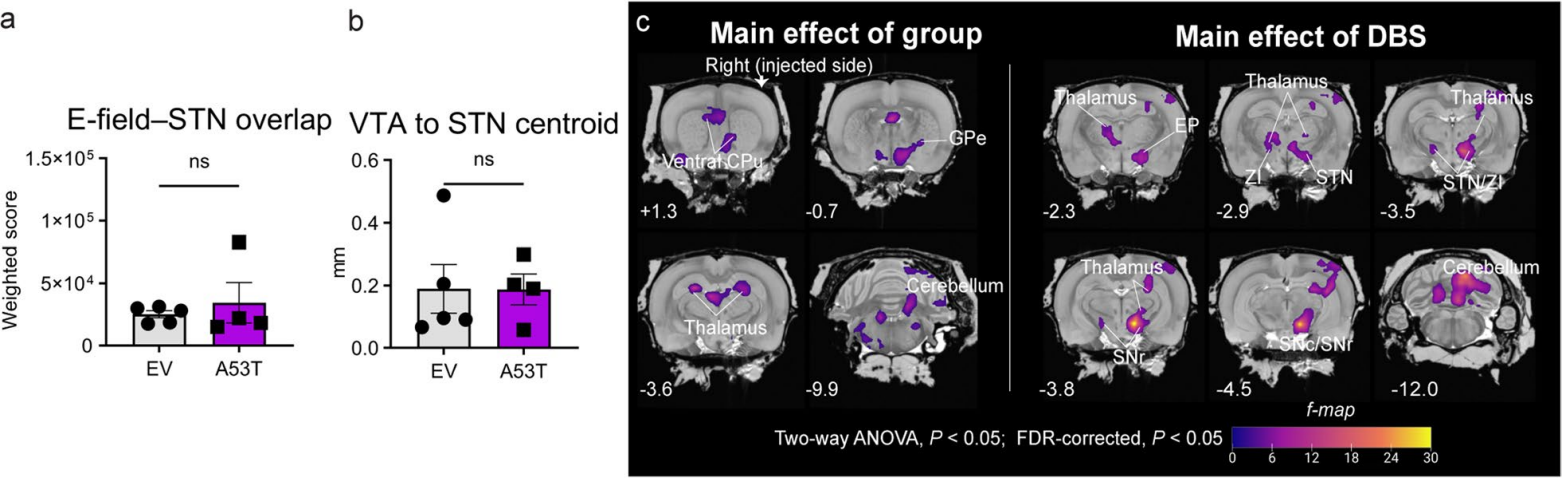
Two-way ANOVA analysis of brain metabolism. **a**, **b** Weighted score of E-field–STN overlap (**a**), and Euclidean distance between the nearest voxel of VTA and STN centroid (**b**) in EV and A53T rats. Data are shown as symbols, mean ± SEM; Mann–Whitney test, ^ns^*P* > 0.05. **c** Significant clusters of main effects (left: group effect; right: DBS effect) after two-way ANOVA analysis (EV OFF/ON, *n* = 5/5, A53T OFF/ON, *n* = 6/4). Clusters are overlaid on a coronal MRI T2 template (Waxholm), with motor regions and y-axis coordinates annotated. Statistical analysis: flexible factorial design with two-way ANOVA (*P* < 0.05), followed by cluster-level FDR correction (*P* < 0.05) in SPM

Although no consistent DBS effects were observed in the STN of EV rats, the comparable weighted score of E-field–STN overlap within the STN (25,174 ± 2907 vs. 34,586 ± 16,156; Mann–Whitney test, *U* = 8, *P* = 0.7302; Fig. 4a) and the VTA location (distance between the nearest VTA voxel and the STN centroid) (0.1894 ± 0.0782 mm vs*.* 0.1875 ± 0.0498 mm; Mann–Whitney test, *U* = 10, *P* > 0.99; Fig. 4b) of the A53T (*n* = 4) and EV (*n* = 5) rats ensured maximized comparability between the two groups. Given this observation, all EV rats (*n* = 5) with DBS were included in ANOVA and behavioral analyses. To identify metabolic alterations in the A53T rats under the OFF-DBS condition (EV vs*.* PD), we employed a two-way ANOVA with post-hoc tests. This approach effectively accounts for both main effects and interactions between group and DBS conditions, providing a robust statistical framework to reveal specific metabolic alterations associated with PD, while distinguishing the effects of DBS.

In the voxel-wise two-way ANOVA analysis, no ‘Group × DBS’ interaction was observed after applying cluster-level FDR correction (*P* < 0.05). However, two significant clusters were identified for the ‘Group’ effect, and 4 clusters were identified for the ‘DBS’ effect (Fig. 4c; Table S2). In the main effect of the ‘Group’, the first cluster did not show significant involvement in motor regions, while the second cluster encompassed motor regions, including the ipsilateral globus pallidus externus (GPe), thalamus (laterodorsal), cerebellum, and bilateral lateral posterior thalamus, as well as the ventral caudate putamen (CPu) (Fig. 4c, Table S2). For the main effect of ‘DBS’, the first 3 clusters demonstrated significant involvement in motor regions, including the ipsilateral entopeduncular nucleus (EP), SNc, and bilateral STN, zona incerta (ZI), substantia nigra pars reticulata (SNr), and thalamus (lateral posterior, posterior and ventral) (Fig. 4c, Table S2).

**Fig. 5:**
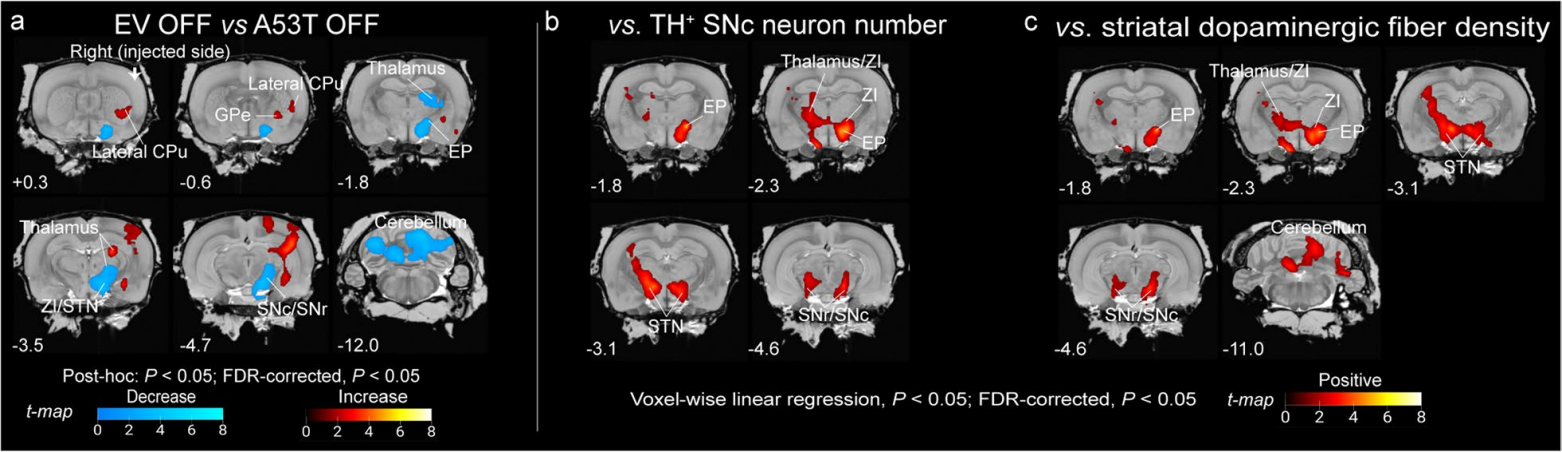
Cerebral metabolic changes in neurodegenerative A53T rats. **a** Significant clusters showing metabolic increases (red voxels, A53T > EV) or decreases (blue voxels, A53T < EV) in A53T rats (DBS OFF, *n* = 6) compared to EV rats (DBS OFF, *n* = 5), overlaid onto a coronal MRI T2 template (Waxholm). Statistical analysis: post-hoc test (*P* < 0.05) followed by cluster-level FDR correction (*P* < 0.05) in SPM. **b** Clusters showing significant correlations (red voxels: positive correlation) of TH^+^ SNc neuron numbers with cerebral metabolism (‘OFF’ images: *n* = 12; A53T: *n* = 7, EV: *n* = 5), overlaid on the coronal MRI T2 template (Waxholm). **c** Clusters showing significant correlations (red voxels: positive correlation) of striatal dopaminergic fiber density with cerebral metabolism (‘OFF’ images: *n* = 12; A53T: *n* = 7, EV: *n* = 5), overlaid on the coronal MRI T2 template (Waxholm). Regression analysis: voxel-wise linear regression (*P* < 0.05), followed by cluster-level FDR correction (*P* < 0.05) in SPM. Motor regions and y-axis coordinates are annotated

To investigate cerebral metabolic changes in A53T rats exhibiting nigrostriatal denervation characteristic of PD-like pathology, we performed a voxel-wise post-hoc analysis following the aforementioned two-way ANOVA, comparing OFF-stimulation A53T rats (*n* = 6) to EV controls (*n* = 5). This approach enabled the identification of brain regions where metabolic activity was altered in A53T rat brains, encompassing both up- and downregulation, thus providing insights into functional changes across the diseased brain. After applying cluster-level FDR correction (*P* < 0.05), three clusters exhibited reduced metabolic activity, while one cluster demonstrated increased metabolic activity (Fig. 5a, Table S3).

The first and dominant cluster showing decreased metabolic activity encompassed brain regions predominantly within the STN–SN/EP–thalamus loop, including the ipsilateral STN, SNc/r, EP, and thalamus (ventral, posterior and laterodorsal) and the ipsilateral ZI (Fig. 5a, Table S3). The second cluster was confined to the cerebellum, exhibiting bilateral effects, while the third cluster did not encompass any motor regions (Fig. 5a, Table S3). The cluster showing increased metabolic activity was limited to the ipsilateral thalamus (lateral posterior), GPe, and lateral CPu (Fig. 5a, Table S3). Subsequent linear regression analyses (‘OFF’ images, *n* = 12; A53T: *n* = 7, EV: *n* = 5) confirmed that hypoactivities in the ipsilateral ZI and STN–SN/EP loop were dependent on the degeneration of the nigrostriatal tract, exhibiting a linear correlation of both the SNc dopaminergic neurons and striatal dopaminergic fibers to the glucose metabolism within the ipsilateral STN, SNc/r, ZI and EP, as well as in the contralateral SNc/r, STN, ZI and thalamus (ventral and posterior) (Fig. 5b, Tables S4–S5). Glucose metabolism in the bilateral cerebellum was found to correlate exclusively with striatal fiber density (Fig. 5c, Table S5). These findings revealed a neurodegeneration-related metabolic pattern in the motor neurocircuit of A53T rats, characterized by prominent hypoactivity within ZI, cerebellum and the STN–SN/EP loop, along with hyperactivity within the lateral CPu–GPe loop.

### STN activation induces functional modulation in A53T rats

**Fig. 6:**
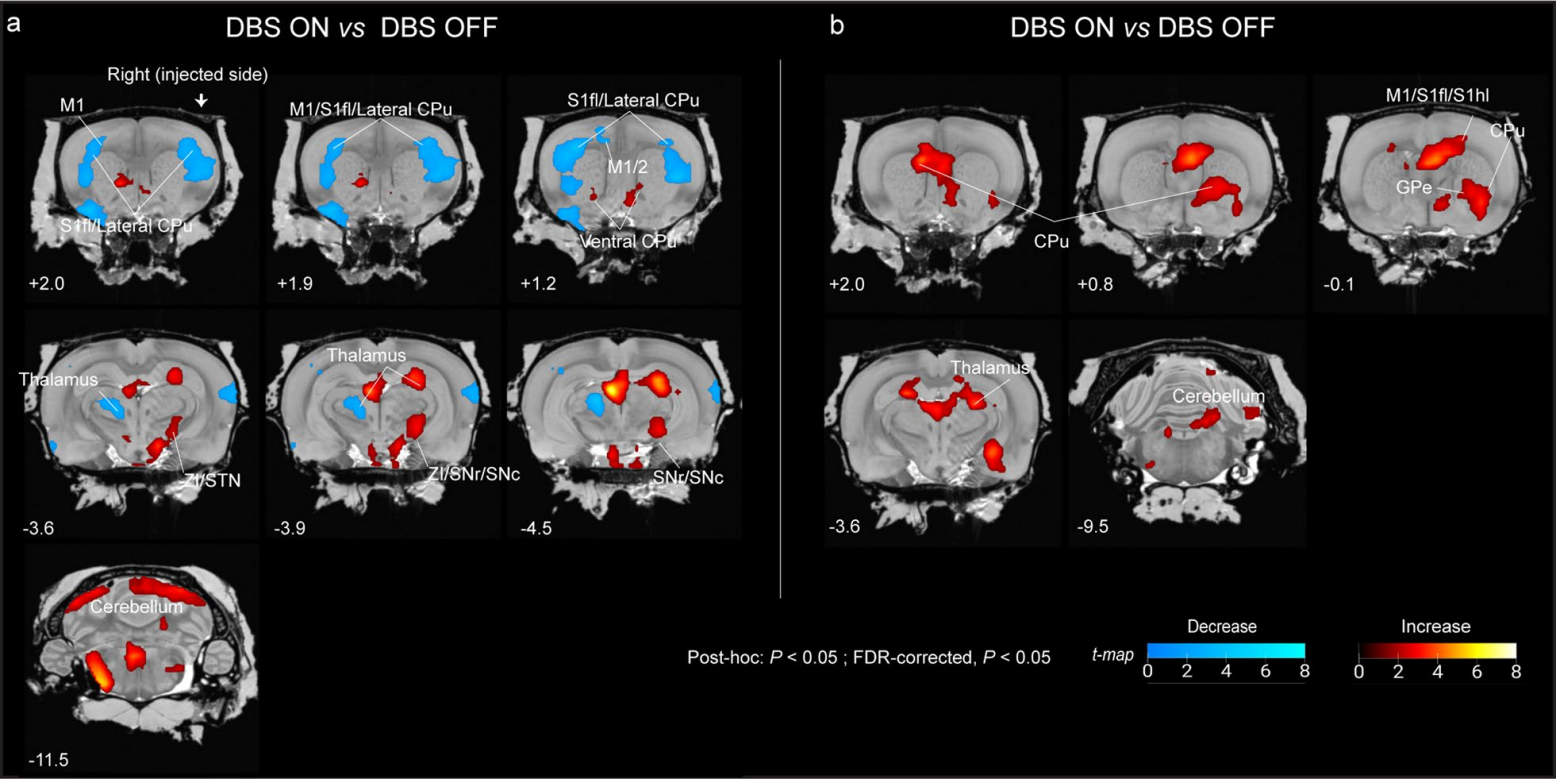
Functional modulation of STN-DBS in A53T and EV rats. **a** Significant clusters showing metabolic increases (red voxels, DBS ON > OFF) or decreases (blue voxels, DBS ON < DBS OFF) in A53T rats (DBS OFF: *n* = 6, DBS ON: *n* = 4) overlaid on the coronal view of the Waxholm MRI T2 template. Statistical analysis: post-hoc test (*P* < 0.05) followed by cluster-level FDR correction (*P* < 0.05) in SPM. **b** Significant clusters showing metabolic increases (red voxels, DBS ON > DBS OFF) or decreases (blue voxels, DBS ON < DBS OFF) in EV rats (DBS OFF: *n* = 5, DBS ON: *n* = 5) overlaid on the coronal view of the Waxholm MRI T2 template. Statistical analysis: post-hoc test (*P* < 0.05) followed by cluster-level FDR correction (*P* < 0.05) in SPM. Motor regions within significant clusters and y-axis coordinates are annotated

To investigate the functional modulation induced by STN activation, we performed a post-hoc test using the aforementioned ANOVA analysis. In the A53T group (DBS OFF: *n* = 6; DBS ON: *n* = 4), following cluster-level FDR correction (*P* < 0.05), we identified 3 significant metabolic clusters (Fig. 6a, Table S6), comprising 2 clusters showing metabolic decreases and one showing an increase. We observed that the STN-DBS upregulated the metabolic activity of ipsilateral regions within the STN–SN–thalamus loop (including the ipsilateral STN, SNc/r, and lateral posterior thalamus) and ZI (Fig. 6a; Table S6). Moreover, DBS significantly increased metabolism in the bilateral cerebellum and ventral CPu (Fig. 6a). Remarkably, STN-DBS led to bilateral hypometabolism in the primary somatosensory area (forelimb representation, S1fl) and lateral CPu, and lateral hypometabolism in the contralateral primary motor area (M1), secondary motor area (M2), and posterior thalamus (Fig. 6a; Table S6).

Intriguingly, unlike the findings in A53T rats, we identified a significant cluster showing increased metabolic activity in the bilateral CPu (both the ventral and lateral part), ipsilateral S1fl, S1hl (hindlimb representation), GPe, lateral posterior/laterodorsal thalamus, and cerebellum of EV rats (DBS OFF: *n* = 5; DBS ON: *n* = 5) (Fig. 6b, Table S7).

These findings suggest that the effects of STN-DBS on the motor circuit in the diseased brains of A53T rats differed from those observed in non-PD EV controls. In A53T rats, STN-DBS activated the target and modulated the brain activity by attenuating regional hypoactivity within the ipsilateral STN–SN loop (Table 1) and deactivating activity in the bilateral cortico-striatal circuitry.

**Table 1:** Metabolic condition in Pakinsonian A53T rats compared to empty AAV1/2 virus (EV) control rats and metabolic changes in response to STN-DBS

Brain region	Ipisilateral to AAV injection		Contralateral to AAV injection	
	Versus EV	Post DBS	Versus EV	Post DBS
Lateral CPu	↑	↓	–	↓
GPe	↑	–	–	–
STN	↓	↑	–	–
ZI	↓	↑	–	–
Thalamus (ventral)	↓	–	–	–
Thalamus (posterior)	↓	–	–	↓
Thalamus (laterodorsal)	↓	–	–	–
SNc	↓	↑	–	–
SNr	↓	↑	–	–
EP	↓	–	–	–
Cerebellum	↓	↑	↓	↑
Thalamus (lateral posterior)	↑	↑	–	–

↑: significant increase; ↓: significant decrease; “–”: no significant change. Significance was defined as *P* < 0.05, followed by cluster-level FDR correction (*P* < 0.05)

### Motor outcomes of STN-DBS

**Fig. 7:**
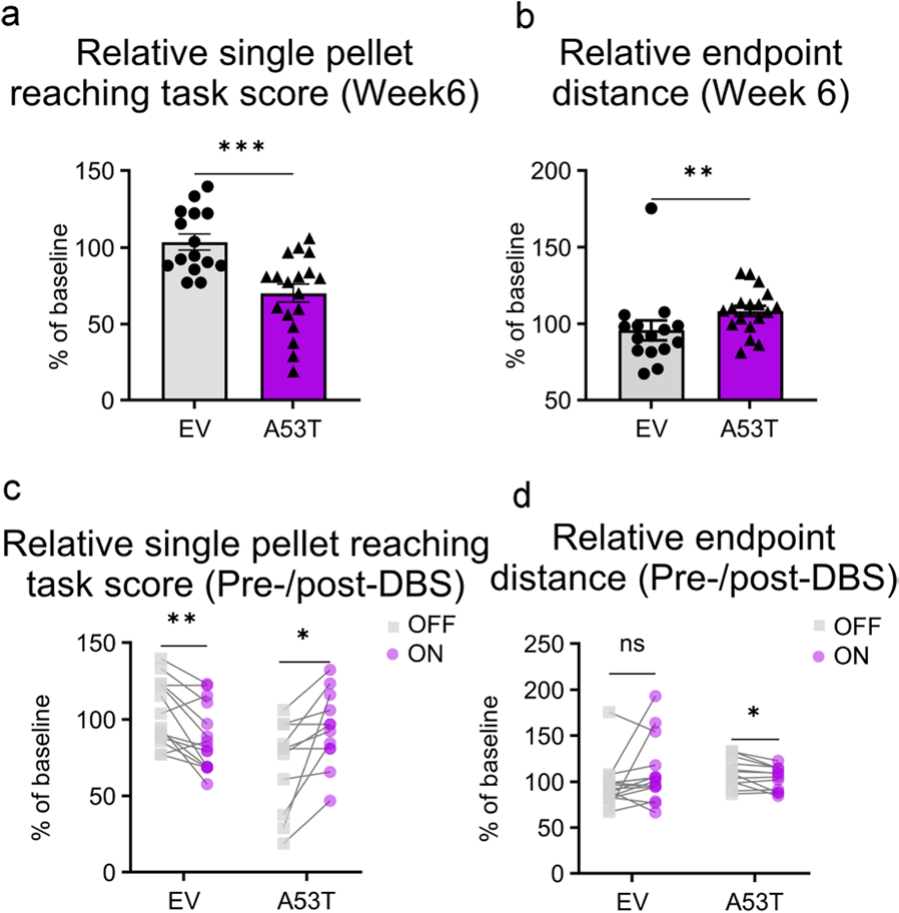
STN-DBS relieves motor dysfunctions in A53T rats. **a**, **b** The relative single pellet reaching task score (**a**) and the relative endpoint distance under OFF-stimulation state (**b**) (EV: *n* = 15 sessions, A53T: *n* = 18 sessions) at week 6. Data are shown as symbols, mean ± SEM; unpaired *t*-test (**a**) or Mann–Whitney test (**b**). **c**, **d** The relative single pellet reaching task score (**c**) and relative endpoint distance (**d**) pre-/post-DBS (EV: *n* = 15 sessions, A53T: *n* = 12 sessions; paired *t*-tests (**c**, and **d**—A53T) or Wilcoxon matched-pairs signed rank test (**d**—EV)). ^*ns*^*P* > 0.05, *P < 0.05, **P < 0.01, ***P < 0.001

To characterize the motor performance of A53T rats 6 weeks after AAV injection, we measured the single pellet reaching task score and reaching kinematics (Fig. S1a). A significant decrease in the relative single pellet reaching task score (% of baseline) was observed in A53T (*n* = 18 sessions) vs. EV rats (*n* = 15 sessions) (70.19% ± 5.873% vs. 103.6% ± 5.343%; unpaired *t*-test, *t*_(31)_ = 4.136, *P* < 0.001; Fig. 7a). In kinematic analyses, compared to the baseline or control group, the density heatmap of A53T rats demonstrated heterogeneity in the end-phase of reaches at week 6 (Fig. S1b–i). To characterize the end-phase parameters of reaching movement, we applied endpoint assessments. Our results uncovered a distant position (% of baseline) from the midpoint of the forepaw to the pellet location (horizontal direction) in A53T rats (*n* = 18 sessions) compared to EV (*n* = 15 sessions) (108.4% ± 3.411% vs. 95.81% ± 6.434%; Mann–Whitney test, *U* = 59, *P* < 0.01; Fig. 7b). The data depicted impaired motor ability in A53T rats, including a reduced single pellet reaching task score and abnormal endpoint control.

To assess the DBS effects on motor function in A53T and EV rats, we compared the relative single pellet reaching task score and relative kinematic parameters during the ON-stimulation state to the OFF-stimulation state. A significant increase in the relative motor score was observed during DBS ON (*n* = 12 sessions) vs. DBS OFF (*n* = 12 sessions) in A53T rats (93.51% ± 6.995% vs. 70.79% ± 8.149%; paired *t*-test, *t*_(11)_ = 3.063, *P* < 0.05; Fig. 7c). Conversely, a significant decline in the relative motor score was found in EV rats after stimulation (*n* = 15 sessions) vs. DBS OFF (*n* = 15 sessions) (87.85% ± 5.502% vs. 103.6% ± 5.343%; paired *t*-test, *t*_(14)_ = 3.758, *P* < 0.01; Fig. 7c). In kinematic analyses, the density heatmap analysis revealed increased homogeneity in the end-phase of reaching movements following stimulation in the A53T rats, whereas no visible changes were observed in EV rats under identical conditions (Fig. S1j–o). Endpoint assessments uncovered a shorter distance (% of baseline) from the midpoint of the forepaw to the pellet location (horizontal direction) in the ON-stimulation (*n* = 12 sessions) vs. the OFF-stimulation (*n* = 12 sessions) A53T rats (103.7% ± 3.734% vs. 110.2% ± 4.541%; paired *t*-test, *t*_(11)_ = 2.58, *P* < 0.05; Fig. 7d). In contrast, no significant difference was observed in EV rats between the ON-stimulation (*n* = 15 sessions) and OFF-stimulation states (*n* = 15 sessions) (109.5% ± 8.967% vs. 95.81% ± 6.434%; Wilcoxon matched-pairs signed rank test, median difference = 6.217, *P* > 0.05; Fig. 7d). These findings confirm the symptomatic benefits of STN-DBS in the A53T rats, including improved behavioral score and endpoint kinematics.

## Discussion

In this study, we integrated reverse translational (patient-to-animal model) methodologies—including three-dimensional electrode, E-field and VTA modeling—into FDG-PET analysis to elucidate the functional modulation induced by unilateral STN-DBS. Spatial registration and normalization using synthetic PET images as a template (PET-to-PET) yield more precise anatomical standardization compared to using an anatomical MR template (PET-to-MR) [23]. We constructed the first FDG-PET template for Sprague-Dawley rats in WHS. The detailed neuroanatomical annotations provided by the WHS atlas [19] allowed us to identify cerebral activity changes and create the mask for region of interest analysis, facilitating a precise interpretation of these changes compared to humans.

Electrode implantation can reduce glucose uptake in the target region [9] and affect the global brain activity [8]. This may significantly impact the comparison with preoperative PD and healthy controls who did not undergo implantation surgery. In our study, we implanted electrodes for both A53T and control rats, and verified electrode localizations using Nissl staining and three-dimensional electrode modeling. In the context of nigrostriatal denervation (~ 60% reduction of dopaminergic neurons in the SNc and ~ 79% reduction in striatal fiber density) in A53T rats, we revealed predominantly hypometabolism in the ipsilateral ZI, cerebellum and the ipsilateral STN–SN/EP–thalamus loop. These significant regions, except for the cerebellum, were linearly correlated with nigrostriatal denervation. The SNc hypoactivity in A53T model is consistent with recent findings in PD patients, which demonstrated a glucose metabolic decline in the midbrain [24–26]. The positive correlation of striatal dopaminergic fiber density with SNc FDG uptake in the A53T model aligns well with patient findings that show a linear correlation of ^18^F-fluoro-*L*-dopa uptake with midbrain glucose metabolism [24]. Notably, we observed that SNc hypoactivity was linked to dopaminergic neuron loss in this brain area. These data indicate that nigral glucose hypometabolism distinguishes PD from healthy controls and can serve as a potential biomarker of neurodegeneration [26].

In A53T rats, the observed STN hypometabolism may result from a reduction in dopaminergic input from the SNc due to neurodegeneration and reduced terminal activity of GABAergic afferents originating from the GPe [27]. The latter is supported by the hypermetabolism observed in lateral CPu and GPe in the current study. This hyperactivity of the GABAergic-mediated striatal-pallidal projection in the pakinsonian state may inhibit the terminal activity of STN afferents, thereby contributing to STN hypometabolism. Notably, electrophysiological recordings in MPTP-treated monkeys demonstrated increased neuronal discharges within the STN [28], which appear to contrast with the hypometabolic STN observed in A53T rats. This discrepancy may, on the one hand, be attributed to inherent methodological differences between microelectrode recordings and FDG-PET imaging. The glucose metabolism—as measured by FDG-PET—reflects integrated synaptic terminal [4] and neural activities [5], whereas neuronal discharge recordings capture the action potentials of a single or a subset of neurons [29]. On the other hand, differences in intrinsic characteristics or in the extent of nigrostriatal system damage between the two models may also account for the observed discrepancy. Further studies are warranted to clarify how neurodegeneration influences STN neuronal firing and its metabolic correlates in the A53T model, thereby advancing our understanding of PD pathophysiology. Additionally, we observed hypometabolism in the ZI, which is a potential DBS target for PD patients [30]. In rats, the main afferents to ZI are GABAergic, originating from the SNc/r, and EP [31]. The hypometabolism observed in the SNc/r and EP could lead to reduced neural activity in ZI afferents, resulting in low metabolism in this area. The decreased metabolic activity of the cerebellum observed in the A53T model contrasts with findings in PD patients [32], but is consistent with results from neurotoxic-based PD models—hypometabolism in the SN/internal pallidum–thalamus and the cerebellum–thalamus pathways in the 6-OHDA-lesioned rats and MPTP-treated monkeys [33].

In line with previous A53T rat model studies [14, 34], our data revealed a reduced relative success rate of single pellet reaching task 6 weeks after A53T viral injection. Furthermore, we observed altered kinematics of reaching movement, characterized by a heterogeneous end-phase trajectory and a distant endpoint location from the target (pellet). These findings revealed poor motor control in neurodegenerative A53T rats. Unilateral STN-DBS effectively ameliorated motor function, as evidenced by an increased behavioral score and improved kinematic parameters after stimulation. The symptomatic benefits of STN-DBS were consistent with reports from PD patients [35–39].

In this study, we employed a fully implantable wireless stimulator [40] and conducted PET scans with and without DBS. Unlike cable-bound stimulation, wireless stimulation allows for continuous stimulation during PET imaging [41]. In CT analyses, we conducted electrode, E-field, and VTA modeling [12]. These methodologies, utilizing the Lead-DBS toolbox [42] and its sister toolbox Lead-Group [43], were translated reversely from patient research. Electrode modeling enables visualization of the electrode position in a three-dimensional view, while E-field and VTA measurements provide parameterized representations of DBS effects in the target area [12]. By leveraging these methods, we uncovered a strong correlation between the ‘nearest voxel of VTA to STN centroid distance’ and changes in FDG uptake or HK1 intensity within the stimulated STN. Specifically, a closer proximity of the VTA to the STN centroid correlated with an upregulation of glucose metabolism. Since HK catalyzes the first step of glycolysis—converting glucose to glucose-6-phosphate—and its expression reflects the rate of glucose metabolism [44], these findings together with the PET-based region of interest analysis confirm that STN-DBS induces local metabolic activation in A53T rats. On the one hand, our results replicate patient findings, demonstrating identical target activation following STN-DBS [9, 37, 45, 46]; on the other hand, we provide a histological validation through HK1 immunofluorescence staining. Activation of the STN area induced upregulation of glucose metabolism in SN areas and exhibited an orthodromic effect. This increased metabolic activity was predominantly concentrated in the pars reticulata of the SN, while it was more sporadically distributed in the pars compacta. Our results support the hypothesis that the SN activity is mediated by the activation of glutamatergic efferents from the STN [47], consistent with the classic basal ganglia model [36, 48]. Moreover, the increased glucose metabolism in the ZI after DBS may result from spreading of the electric field [49], as the ZI is located in the vicinity of the STN in rats. In contrast, STN-DBS in the EV rats resulted in a distinct metabolic pattern in basal ganglia, characterized by activated glucose uptake in the GPe and CPu. Interestingly, a similar metabolic upregulation in the cerebellum observed in both groups suggests that cerebellar modulation may not be specific to A53T rats. Thus, we propose that STN-DBS exerts a specific activation of the STN–SN loop of A53T rats, predominantly causing local activation and further promoting orthodromic activation in connected regions. These effects ameliorate the hypometabolism of the STN–SN pathway observed in OFF-stimulation A53T rats. By characterizing functional alterations in the Parkinsonian state, we provide critical context for interpreting the effects of STN-DBS in PD. We observed remote deactivation in the bilateral cortex and CPu following STN-DBS in A53T rats, in contrast to EV rats. Specifically, cortical deactivation was identified in the bilateral S1fl, M1, and contralateral M2. It has been hypothesized that reduced cortical metabolism is driven by enhanced basal ganglia–thalamus inhibitory output and subsequent reduced activity in thalamocortical projections [36, 46]. However, we did not observe significant metabolic decline in the ipsilateral thalamus of stimulated A53T rats. Thus, the classic circuit model [50] might be insufficient to explain our PET findings and motor improvements after DBS. Alternatively, our data support the hypothesis of DBS-induced antidromic cortical modulation via the hyperdirect pathway, as previously suggested in STN-DBS studies using 6-OHDA rodent models [51, 52]. Of note, the metabolic downregulation in the CPu following STN-DBS was predominantly localized to the lateral part and is likely mediated by reduced cortical activity via excitatory projections from both M1 and S1 [53]. Interestingly, a similar pattern of metabolic deactivation emerged in the contralateral cortical areas (M1, M2, S1fl) and lateral CPu. This bilateral metabolic modulations likely result from interhemispheric functional interactions [54] or antidromic modulation of STN afferents from the bilateral cortex [55]. Intriguingly, STN-DBS exhibited differential regulation across CPu subregions, characterized by increased glucose metabolism in the ventral CPu and decreased metabolism in the lateral CPu. While the underlying mechanisms remain unclear, these findings may reflect region-specific variations in CPu responsiveness to STN-DBS, warranting further investigation. Studies have shown reduced metabolic activity in cortical regions and/or the CPu but these studies used bilateral STN-DBS [36, 39, 46, 48]. For instance, bilateral STN-DBS reduced glucose metabolism in the putamen and sensorimotor cortex [46]. Our findings bridge the gap in understanding how unilateral STN-DBS affects the bilateral cortico-striatal circuitry, offering insights into the mechanisms underlying the bilateral motor relief induced by unilateral STN-DBS in PD patients [56].

Notably, the effects of unilateral STN-DBS are not confined to the local brain area (STN) but extend remotely and bilaterally, indicating a brain-wide neuromodulation. Recently, an STN-DBS-specific spatial covariance pattern—StimNet—was identified in PD patients using FDG-PET imaging [57]. The metabolic-based StimNet expression after STN-DBS—such as increased metabolism in the STN and SN, and decreased metabolism in the sensorimotor cortex—are consistent with the alterations observed in the A53T model [57]. Although our results show strong comparability with patient observations, certain limitations should be acknowledged. First, while patient-mimicking pathological changes—such as nigrostriatal degeneration and Lewy body accumulation—along with motor deficits, have been well characterized in the A53T rat model [13, 14], their responsiveness to levodopa, a criterion for PD patient selection for DBS therapy [58], has not yet been assessed. Second, the aforementioned stimNet expression during the OFF-stimulation state and its changes following STN-DBS were found to relate to motor improvements [57]. In our study, we did not directly assess correlations between the metabolic pattern and behavioral outcomes due to the limited sample size. Nonetheless, the similarity in the spatial distribution and directionality of these changes supports the utility of the A53T model in capturing DBS-induced functional modulation observed in PD patients. One of the aims of this work was to reverse-translate patient-driven approaches into a PD rat model, which inherently confers an exploratory nature. Future studies with larger cohorts are warranted to investigate the relationship between DBS-induced metabolic alterations and motor outcomes. Third, although we allowed a 14-day recovery period after electrode implantation, we cannot fully rule out the possibility that residual surgical or microlesion effects have influenced the observed outcomes. To minimize the impact of surgery and implantation-related microlesion effects between groups, we implanted electrodes in both A53T and EV rats. Moreover, the electrodes were designed with a small diameter (100 µm) and a fine, tapered tip to further reduce local tissue disruption. Fourth, although we employed a sensitive behavioral test—the single pellet reaching task—to evaluate forepaw motor function in rats [59] and complemented our analysis with a deep learning–based approach (DeepLabCut) to characterize motor kinematics as comprehensively as possible, these assessments cannot fully capture the spectrum of motor deficits observed in PD patients due to the inherent species differences. Additional motor assessments, such as the open field, cylinder, or rotarod tests, should be incorporated in future studies to provide a more comprehensive evaluation of motor function in PD rodent models.

## Conclusions

Collectively, our study provides a comprehensive characterization of circuit-level modulations induced by unilateral, therapeutic STN-DBS in the A53T PD rat model. We conclude that: (1) the STN–SN/EP–thalamus hypometabolism is predominantly exhibited in A53T rats compared to EV controls; (2) therapeutic STN-DBS activates the target region and ameliorates the aberrant metabolic activities in the ipsilateral STN–SN loop; and (3) STN-DBS leads to deactivation of brain regions within the bilateral cortico-striatal circuitry. We underscore the utility of reverse translational methodologies in revealing local and global modulation underlying therapeutic STN-DBS in an A53T PD rat model. The integration of molecular imaging and parameterized DBS field effect characterization, enhances comparability between clinical and basic research, and facilitates data integration across studies and research centers. This pipeline offers a significant advantage by preserving brain tissue integrity, as it eliminates the need for staining focused solely on electrode trajectory identification. This strategy supports more downstream analyses, such as investigating complementary molecular and cellular mechanisms of DBS.

## Supplementary Information

Additional file 1. **Supplementary materials and methods**. **Table S1**. Electrode tip properties and DBS parameters. **Table S2**. Two-way ANOVA results in SPM. **Table S3**. Metabolic changes in motor regions of A53T rats (vs. EV). **Table S4**. Voxel-wise linear regression with TH^+^ SNc neuron number. **Table S5**. Voxel-wise linear regression with striatal dopaminergic fiber density. **Table S6**. Motor regions affected by STN-DBS in A53T rats. **Table S7**. Motor regions affected by STN-DBS in EV rats. **Fig. S1**. Trajectory and density heatmap analyses of single pellet reaching task.

## Data Availability

The datasets used and/or analysed during the current study are available from the corresponding author on reasonable request.

## Abbreviations

CPu: Caudate putamen
DAPI: 4′,6-Diamidino-2-phenylindole
E-field: Electric field
EP: Entopeduncular nucleus
EV: Empty vector
FDG-PET: ^18^F-fluorodeoxyglucose PET
FDR: False discovery rate
GPe: Globus pallidus externus
HK: Hexokinase
M1/2: Primary/secondary motor area
PD: Parkinson's disease
P_un-corr_: Uncorrected *p* value
q_FDR-corr_: Quantile FDR corrected *p* value
S1fl: Primary somatosensory area, forelimb representation
S1hl: Primary somatosensory area, hindlimb representation
SNc/r: Substantia nigra pars compacta/reticulata
SPM: Statistical parametric mapping
STN: Subthalamic nucleus
STN-DBS: Subthalamic deep brain stimulation
SUVr: Standardized uptake value ratio
TH^+^: Tyrosine hydroxylase-positive
VTA: Volume of tissue activated
WHS: Waxholm space
ZI: Zona incerta
α-syn: Alpha-synuclein
