# Supplementary material for: Molecular glucose imaging reveals functional brain reconfiguration by subthalamic deep brain stimulation in Parkinsonian rats
Source: Transl Neurodegener. 2025 Dec 2;14:62. doi: 10.1186/s40035-025-00523-3 (PMC12670866; doi:10.1186/s40035-025-00523-3)
Supplement: Supplementary file 1 — Additional file 1. Supplementary materials and methods. Table S1. Electrode tip properties and DBS parameters. Table S2. Two-way ANOVA results in SPM. Table S3. Metabolic changes in motor regions of A53T rats (vs. EV). Table S4. Voxel-wise linear regression with TH+ SNc neuron number. Table S5. Voxel-wise linear regression with striatal dopaminergic fiber density. Table S6. Motor regions affected by STN-DBS in A53T rats. Table S7. Motor regions affected by STN-DBS in EV rats. Fig. S1. Trajectory and density heatmap analyses of single pellet reaching task. [file 40035_2025_523_MOESM1_ESM.docx]

# Supplementary information

Molecular glucose imaging reveals functional brain reconfiguration by subthalamic deep brain stimulation in Parkinsonian rats

*Jiazhi Chen, Ningfei Li, Muthuraman Muthuraman, Nengxing Liang, Jens Volkmann, Takahiro Higuchi, Chi Wang Ip*

Corresponding author: Chi Wang Ip, [ip_c@ukw.de](mailto:ip_c@ukw.de)

**This file includes:**

Supplementary materials and methods.

Supplementary figure S1 and legends.

Supplementary tables S1–S7 and legends.

# Supplementary materials and methods

# Single pellet reaching task

## Training

Rats were trained in a Plexiglas box (34×14 cm) equipped with an opening and a platform on one side to reach and grasp food pellets with the forepaw. During the initial 3 days of training, the dominant forepaw of each rat was identified. Subsequently, the rats were exclusively trained to use their dominant forepaw. This training was conducted for 10 minutes per day over a period of 16 days until proficient reach and grasp behaviors were established. After the baseline recording, training sessions were conducted twice a week to ensure reproducibility.

## Recording

To reduce variability in behavioral assessments, three recordings were obtained for each recording time point, with sessions conducted continuously over 3 days (one session per day). Each session was recorded for 7.5 minutes using a digital side-view camera operating at a high frame rate of 120 fps. For recording sessions at week 6 after AAV injection, single pellet reaching task recordings were initially performed without Subthalamic deep brain stimulation (STN-DBS). Subsequently, the stimulators were activated for 5 minutes, and the same single pellet reaching task recording procedures were repeated.

## Visual scoring

The first 20 trials of each recording session were assessed. A score of 1 indicated a successful grasp during the first reach, with immediate delivery of the pellet to the mouth. A score of 0.5 was assigned if the pellet was dropped into the chamber ground before consumption, while a score of 0 corresponded to a failed grasp. The total score for each animal in each session was calculated by summing individual trial scores. The relative single pellet reaching task score was used to evaluate motor performance and calculated as: *Relative score (% of baseline)* *= (session score_week6/_mean score_baseline_)×100*. Rats with stable success rates above 40% during baseline sessions (1) were included in the subsequent behavioral analyses.

## Posture estimation using DeepLabCut

To mitigate the influence of irrelevant behaviors such as grooming, scratching, or chewing, all forepaw-reaching movements were identified, extracted, and subsequently combined through concatenation in sequential order.

The start of a reaching movement was defined as the moment when the forepaw began to move forward (2). The end of a reaching movement was defined as the moment when the forepaw either successfully retrieved the pellet or withdrew after an unsuccessful grasp. For posture estimation, we employed DeepLabCut as outlined in the works of A. Mathis (3) and T. Nath (4). The training dataset consisted of ten videos, from which 24 frames were extracted per video, resulting in a dataset of 240 frames. Frame selection utilized the "*k-means*" and "*uncertain*" functions inherent to DeepLabCut. During the labeling phase, we annotated 17 body parts, five structures within the behavioral recording chamber, and the pellet. The neural architecture employed was based on ResNet-50, using default parameters. A total of 550,000 training iterations were executed to optimize the network performance. Validation involved employing a specific number of shuffles, with the test error quantified at 10.12 pixels for the test set and 4.01 pixels for the training set (video size at 640×480 pixels), respectively. To streamline subsequent analyses, a p-cutoff threshold of 0.6 was applied to condition the x and y coordinates. Subsequently, this trained network was deployed for the analysis of videos derived from the same recording configurations.

**Kinematic analyses**

Kinematic analyses were performed using custom-written scripts in MATLAB (Version 2019b, Mathworks, Natick, MA, USA). The estimated likelihood from DeepLabCut was first subjected to a high-pass filter (*adp_filt* function from the B-SOID toolbox), to replace data-driven low-likelihood positions with the most recent highly probable position (5). Additionally, the x and y coordinates were recalibrated relative to the food pellet position, designating it as the origin ([0,0]). To enable direct comparisons between the horizontally flipped videos of left-handed and the right-handed behavioral videos across subjects and groups, an additional normalization step was implemented. This normalization process involved dividing each pixel value by the total pixel count, spanning from the lower-left corner to the lower-right corner of the platform. The resulting normalized values were then expressed in arbitrary units, providing a standardized basis for subsequent kinematic computations.

The ‘reach’ trajectory during the single pellet reaching task was determined using the midpoint of the forepaw as the reference point. The MATLAB peak algorithm was utilized to identify the maximum distance in the horizontal direction. The endpoint of a ‘reach’ was defined as the first coordinate where the forepaw reached the maximum distance in the outward direction (6). Only reaching trajectories with all estimated coordinates exceeding a likelihood threshold of 0.6 were considered qualified trials, and were included in the analysis. To obtain the average trajectory for all single reaches within the group and facilitate accurate comparisons of reaching patterns across groups or conditions, the x-coordinates of raw trajectories were interpolated to ensure consistent scale. To visualize the spatial distribution of reaching movements, density heatmaps were generated.

The endpoint assessments were utilized to evaluate the motor function of the skilled reaching behaviors (6, 7). The ‘reach’ trials utilized for trajectory analysis were then employed for endpoint assessment. The Euclidean distance in the horizontal direction to the pellet was computed by subtracting the x-coordinate of the endpoint location in the hand midpoint from the x-coordinate of the pellet location. To ensure consistency across videos with differing numbers of qualified reaches, we included the initial ten trials for subsequent statistical analysis. If a video contained fewer than ten qualified trials, all qualified trials were incorporated. The mean distance was calculated for each recording session. The relative endpoint distance was determined as a percentage: *relative endpoint distance (% of baseline)=(mean distance of each session_wk6_ /mean distance_baseline_)×100.*

# Histology and immunohistochemistry

## Tissue processing

Animals were stimulated for 30 minutes to induce acute metabolic changes in the subthalamic nucleus (STN). Sixty minutes after stimulation, the animals were euthanized using CO_2_ and subsequently perfused transcardially with ~120 mL of 0.1 mol/L phosphate-buffered saline solution (PBS) containing heparin, followed by ~120 mL of 4% paraformaldehyde in 0.1 mol/L PBS until the liver was thoroughly cleaned. The whole brains were then post-fixed in 4% paraformaldehyde in 0.1 mol/L PBS solution for 1 day and subsequently dehydrated in 30% sucrose in 0.1 mol/L PBS for another 4 days. Brains were frozen in liquid dry ice-cooled isopentane. Tissues were then serially sectioned coronally at 40 µm thickness and collected sequentially in six series for the substantia nigra pars compacta (SNc) sections and 12 series for the striatal sections. All sections were stored in an anti-freezing solution at -20°C.

## Tyrosine Hydroxylase (TH)-Nissl and Nissl staining

Free-floating SNc or striatal sections were initially incubated in a solution containing 10% normal goat serum, 2% bovine serum albumin, and 0.5% Triton X-100 in 0.1 mol/L PBS for 1 hour. Subsequently, the sections were immunostained overnight at room temperature with primary rabbit anti-rat TH antibody (1:500 for the striatum, 1:1000 for the SNc; Cat.#ab112; Abcam, Cambridge, UK). Following immunostaining, the sections were washed 3 times for 5 minutes each in 0.1 mol/L PBS. A biotinylated goat anti-rabbit antibody (1:100 for both striatum and SNc; Cat.#BA-1000; Vector Laboratories, Burlingame, CA, USA) was applied for 2 hours, followed by incubation with avidin/biotin reagent (Cat.#32050; Thermo scientific, USA) and DAB (Cat.#SK-4100; Vector Laboratories, Burlingame, CA, USA). Nissl staining was performed on SNc sections. After washing in 0.1 mol/L PBS, SNc sections were mounted on object slides and dried at room temperature. The sections were incubated in cresyl violet solution (1 g of cresyl violet dissolved in 10 mL of 100% acetic acid and diluted to 1 liter with distilled water) for 30 minutes at room temperature. After washing 3 times in distilled water for 5 minutes each, the sections were dehydrated in an ascending ethanol series (70%, 96%, and 100%), then incubated twice in xylene solution for 5 minutes each. Sections were then covered with coverslips. Same Nissl staining protocol was implemented on free-floating STN sections for electrode trajectory identification histologically.

## Immunofluorescence staining

### TH/alpha-synuclein (α-syn)/4’,6-diamidino-2-phenylindole (DAPI)

Free-floating SNc sections were first blocked with 10% normal goat serum and 2% bovine serum albumin for 1 hour at room temperature, followed by overnight incubation (4°C) in primary chicken-anti-TH (1:30000; Cat.#ab76442; Abcam, Cambridge, UK) and rabbit-anti-human α-syn-antibodies (1:500; Cat.#S3062; Sigma, Sigma-Aldrich, St Louis, MO, USA). Sections were incubated with goat anti-chicken Alexa Fluor 488 (1:300; Cat.#A11039; Invitrogen, Frederick, MD) and goat-anti-rabbit Cy3 secondary antibodies (1:300; Cat.#111-165-144; Jackson ImmunoResearch Laboratories Inc., West Grove, PA) for 2 hours at room temperature on the second day. DAPI nuclear staining (1:500; Cat.#D8417; Sigma, Sigma-Aldrich, St Louis, MO, USA) was performed at room temperature for 20 minutes. The sections were mounted onto object slides and covered with coverslips using Aqua Polymount (Cat.#18606; Polysciences, Warrington, PA).

### Hexokinase (HK) 1/NeuN/DAPI immunofluorescence staining

Free-floating STN sections were first quenched in 100 mmol/L glycine (Cat.#G8898; Sigma; Sigma-Aldrich, St Louis, MO, USA) for 1 hour at room temperature, followed by a blocking step using a solution containing 10% normal donkey serum, 2% bovine serum albumin, 0.3% Triton X-100, and 0.1% Tween-20 in 0.1 mol/L PBS for 1 hour at room temperature. Sections were then incubated overnight at 4°C with primary pig anti-NeuN (1:400; Cat.#226004; Synaptic Systems GmbH, DE) and mouse anti-HK1-antibodies (1:1000; Cat.#MAB1532; Sigma, Sigma-Aldrich, St Louis, MO, USA). Secondary donkey anti-pig Cy5 (1:300; Cat.#706-175-148; Jackson ImmunoResearch Laboratories, Inc., West Grove, PA) and donkey anti-mouse Cy3 secondary antibodies (1:300; Cat.#715-166-150; Jackson ImmunoResearch Laboratories, Inc., West Grove, PA) were applied for 2 hours at room temperature on next day. Nuclear staining was performed using DAPI (1:500; Cat.#D8417; Sigma; Sigma-Aldrich, St Louis, MO, USA) for 20 minutes at room temperature. Finally, sections were cover-slipped with Aqua Polymount (Cat.#18606; Polysciences, Warrington, PA).

## Unbiased stereology of TH^+^/Nissl^+^ SNc cells

The Stereo Investigator software (Version 11.07; MicroBrightField Biosciences, Williston, VT) was used. Seven to eight SNc sections were analyzed, spaced 240 µm apart. Key parameters included a grid size of 130×130 µm, a counting frame of 60×60 µm, and a 1.5 µm guard zone. Actual section thickness was determined by measuring randomly selected sections. Sections were examined using a BX53 microscope with a 100×/1.25 numerical aperture objective lens (Olympus, Tokyo, Japan). Gundersen coefficients of error for m=1 were all ≤0.10, ensuring reliable counting.

## Striatal dopaminergic fiber density

The optical density of TH^+^ dopaminergic fibers in the striatum was quantified using the NIH ImageJ software. Representative pre-commissural, commissural and post-commissural striatum sections were included for optical density quantification. The process involved converting color images into 8-bit grayscale format and normalizing the ipsilateral background-corrected striatal area to the contralateral background-corrected side using the formula: *(ipsilateral optical density-optical density_Corpus callosum_*)*/(contralateral optical density*-*optical density_Corpus callosum_)×100*. The mean optical density value was calculated for each animal.

## Normalized HK1 intensity

HK1 signal intensity in the central STN (-3.6 mm anteroposterior) was measured using NIH ImageJ software. The STN border was first delineated based on the NeuN channel. To normalize for background and inter-animal variability, three reference regions (each 0.11 × 0.11 mm) were evenly selected within the internal capsule ipsilaterally. The normalized HK1 intensity was calculated by dividing the mean HK1 signal intensity in the STN by the mean intensity of the selected reference areas.

**Table S1 Electrode tip properties and DBS parameters.**

| **Rats** | **Fiber material** | **Tip impedance (kΩ)** | **Surface area of exposed metal (cm^2^)** | **DBS current (µA)** | **Pulse width (µs) /Frequency (Hz)** |
| --- | --- | --- | --- | --- | --- |
| EV 01 | 95% platinum  5% tungsten  Iridium oxide coated | ~100 | ~1.5×10^-5^ (before Iridium oxide coating) | 110 | 60 /130 |
| EV 02 |  |  |  | 120 |  |
| EV 03 |  |  |  | 100 |  |
| EV 04 |  |  |  | 100 |  |
| EV 05 |  |  |  | 100 |  |
| A53T 01 |  |  |  | 60 |  |
| A53T 02 |  |  |  | 70 |  |
| A53T 03 |  |  |  | 95 |  |
| A53T 04 |  |  |  | 70 |  |
| A53T 05 |  |  |  | 85 |  |
| A53T 06 |  |  |  | 60 |  |
| A53T 07 |  |  |  | 70 |  |

**Table S2** Two-way ANOVA results in SPM

| **Effects** | **Motor regions** | **Clusters** | **Cluster size** | **q_FDR-corr_** | ***P*_un-corr_** |
| --- | --- | --- | --- | --- | --- |
| Group × DBS | - | - | - | > 0.05 | > 0.05 |
| Group | - | Cluster 1 | 7260 | **< 0.001** | **< 0.001** |
|  | **Right:** GPe, Thalamus (laterodorsal), Cerebellum **Bilateral:** Ventral CPu, Thalamus (lateral posterior ) | Cluster 2 | 19689 | **< 0.001** | **< 0.001** |
| DBS | **Left:** Thalamus (lateral posterior)  **Right:** EP, SNc  **Bilateral:** STN, ZI, SNr, Thalamus (posterior), Thalamus (ventral) | Cluster 1 | 2637 | **0.016** | **0.001** |
|  | **Bilateral:** Cerebellum | Cluster 2 | 5814 | **< 0.001** | **< 0.001** |
|  | **Right:** Thalamus (lateral posterior), Cerebellum | Cluster 3 | 5822 | **< 0.001** | **< 0.001** |
|  | - | Cluster 4 | 2612 | **0.016** | **0.001** |

Statistical profile of all significant clusters identified from the two-way ANOVA in SPM.

| **Table S3 Metabolic changes in motor regions of A53T rats (vs*.* EV)** | | | | | |
| --- | --- | --- | --- | --- | --- |
| **Effects** | **Motor regions** | **Clusters** | **Cluster size** | **q_FDR-corr_** | ***P*_un-corr_** |
| Decrease | **Right**: STN, ZI, Thalamus (ventral), SNc, SNr, EP, Thalamus (posterior), Thalamus (laterodorsal) | Cluster 1 | 4744 | **0.029** | **0.001** |
|  | **Bilateral**: Cerebellum | Cluster 2 | 9213 | **0.001** | **< 0.001** |
|  | - | Cluster 3 | 3772 | **0.049** | **0.003** |
| Increase | **Right**: Lateral CPu, GPe, Thalamus (lateral posterior) | Cluster 1 | 9319 | **0.001** | **< 0.001** |

Statistical profile of significant clusters obtained through the SPM analysis comparing EV (DBS OFF) and A53T (DBS OFF).

| **Table S4 Voxel-wise linear regression with TH^+^ SNc neuron number** | | | | | |
| --- | --- | --- | --- | --- | --- |
| **Correlation** | **Motor regions** | **Clusters** | **Cluster size** | **q_FDR-corr_** | ***P*_un-corr_** |
| Positive | **Left**: Thalamus (posterior, ventral) **Right**: EP **Bilateral**: STN, SNr, SNc, ZI | Cluster 1 | 5972 | **0.027** | **0.001** |

Statistical profile of significant clusters observed through the voxel-wise linear regression between cerebral metabolism and TH^+^ SNc neuron number.

| **Table S5 Voxel-wise linear regression with striatal dopaminergic fiber density** | | | | | |
| --- | --- | --- | --- | --- | --- |
| **Correlation** | **Motor regions** | **Clusters** | **Cluster size** | **q_FDR-corrr_** | ***P*_un-corr_** |
| Positive | **Left**: Thalamus (posterior, ventral) **Right**: EP **Bilateral**: STN, SNr, SNc, ZI | Cluster 1 | 5883 | **0.026** | **0.001** |
|  | **Bilateral**: Cerebellum | Cluster 2 | 5169 | **0.026** | **0.001** |

Statistical profile of significant clusters extracted from the voxel-wise linear regression between cerebral metabolism and striatal dopaminergic fiber density.

| **Table S6 Motor regions affected by STN-DBS in A53T rats** | | | | | |
| --- | --- | --- | --- | --- | --- |
| **Effects** | **Motor regions** | **Clusters** | **Cluster size** | **q_FDR-corr_** | ***P*_un-corr_** |
| Increase | **Right**: STN, ZI, Thalamus (lateral posterior), SNr, SNc  **Bilateral**: Ventral CPu, Cerebellum | Cluster 1 | 27504 | **< 0.001** | **< 0.001** |
| Decrease | **Right**: M1, Lateral CPu, S1fl | Cluster 1 | 8442 | **0.002** | **< 0.001** |
|  | **Left**: M1, M2, S1fl, Lateral CPu, Thalamus (posterior) | Cluster 2 | 10442 | **0.001** | **< 0.001** |

Statistical profile of all significant clusters identified in the SPM analysis of DBS effects on A53T rats.

| **Table S7 Motor regions affected by STN-DBS in EV rats** | | | | | |
| --- | --- | --- | --- | --- | --- |
| **Effects** | **Motor regions** | **Clusters** | **Cluster size** | **q_FDR-corr_** | ***P*_un-corr_** |
| Increase | **Right**: GPe, Thalamus (lateral posterior), Thalamus (laterodorsal), M1, S1fl S1hl, Cerebellum **Bilateral**: CPu | Cluster 1 | 21026 | **< 0.001** | **< 0.001** |

Statistical profile of all significant clusters identified in the SPM analysis of DBS effects on EV rats.

**
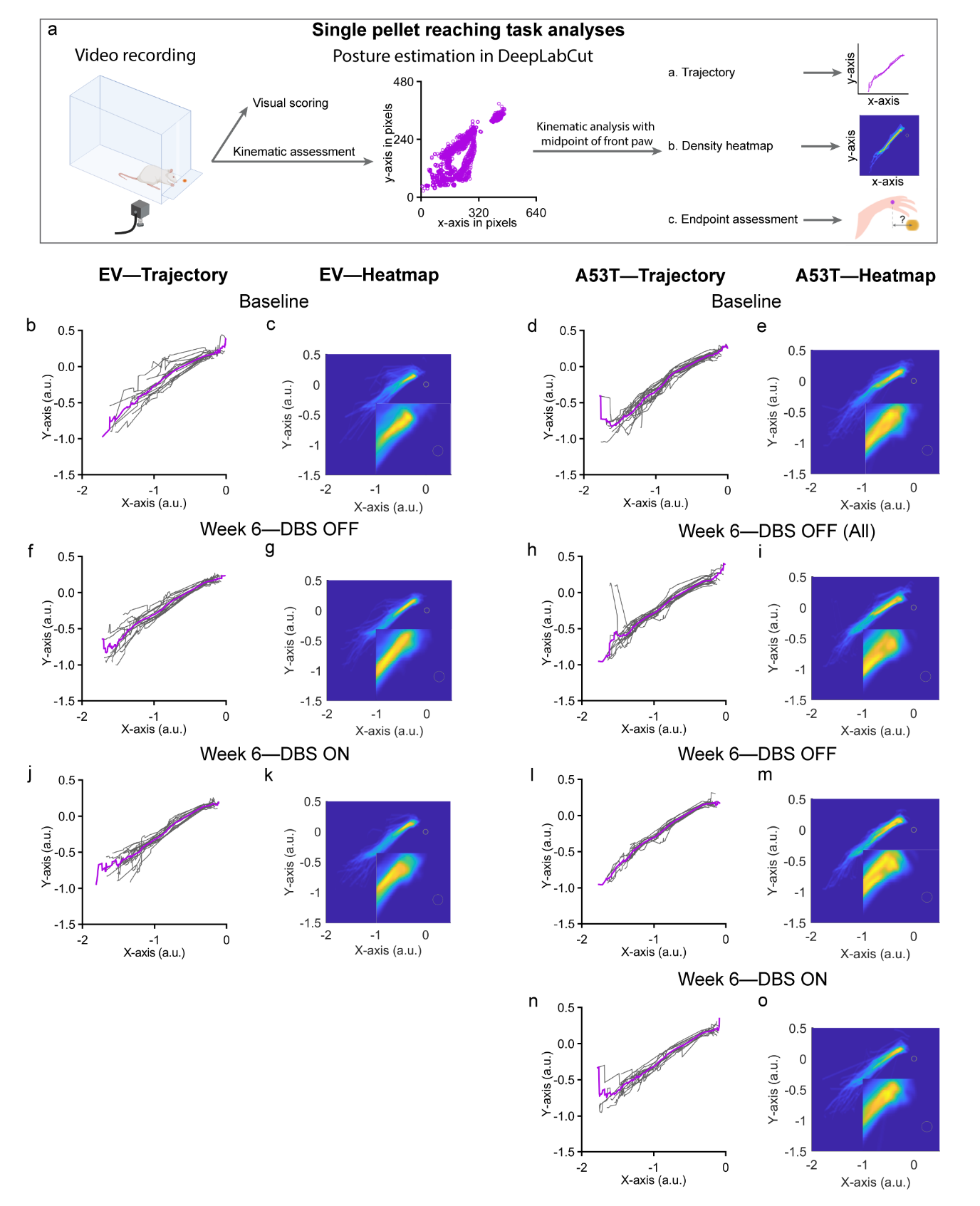
**

**Fig. S1** **Trajectory and density heatmap analyses of single pellet reaching task. a** Single pellet reaching task analyses pipeline. **b-i** Reaching trajectories and corresponding density heatmaps at baseline (**b-c**, EV, *n*=15 sessions; **d-e**, A53T, *n*=18 sessions) and week 6 (**f-g**, EV, *n*=15 sessions; **h-i**, A53T, *n*=18 sessions). **j-k** Reaching trajectories and corresponding density heatmaps post-DBS in the EV group (*n*=15 sessions) at week 6. **l-o** Reaching trajectories and corresponding density heatmaps pre-DBS (**l-m**, *n*=12 sessions) and post-DBS (**n-o**, *n*=12 sessions) in the A53T group at week 6. Individual trajectories are shown in grey, and the mean trajectories are shown in pink. A magnification of the end-phase is located in the lower right of each heatmap, and the pellet location is illustrated in a grey circle.

# References

1. Bova A, Gaidica M, Hurst A, Iwai Y, Hunter J, Leventhal DK. Precisely timed dopamine signals establish distinct kinematic representations of skilled movements. Elife. 2020;9.

2. Bova A, Ferris K, Leventhal DK. Evolution of gross forelimb and fine digit kinematics during skilled reaching acquisition in rats. eNeuro. 2021;8(5).

3. Mathis A, Mamidanna P, Cury KM, Abe T, Murthy VN, Mathis MW, et al. DeepLabCut: markerless pose estimation of user-defined body parts with deep learning. Nat Neurosci. 2018;21(9):1281-9.

4. Nath T, Mathis A, Chen AC, Patel A, Bethge M, Mathis MW. Using DeepLabCut for 3D markerless pose estimation across species and behaviors. Nat Protoc. 2019;14(7):2152-76.

5. Hsu AI, Yttri EA. B-SOiD, an open-source unsupervised algorithm for identification and fast prediction of behaviors. Nat Commun. 2021;12(1):5188.

6. Becker MI, Person AL. Cerebellar control of reach kinematics for endpoint precision. Neuron. 2019;103(2):335-48 e5.

7. Lopez-Huerta VG, Denton JA, Nakano Y, Jaidar O, Garcia-Munoz M, Arbuthnott GW. Striatal bilateral control of skilled forelimb movement. Cell Rep. 2021;34(3):108651.
